# Clinical phenotyping and genetic diagnosis of a large cohort of Sudanese families with hereditary spinocerebellar degenerations

**DOI:** 10.1038/s41431-023-01344-6

**Published:** 2023-04-03

**Authors:** Ashraf Yahia, Ahlam A. A. Hamed, Inaam N. Mohamed, Maha A. Elseed, Mustafa A. Salih, Sarah M. El-sadig, Hassab Elrasoul Siddig, Ali Elsir Musa Nasreldien, Mohamed Ahmed Abdullah, Maha Elzubair, Farouk Yassen Omer, Aisha Motwakil Bakhiet, Rayan Abubaker, Fatima Abozar, Rawaa Adil, Sara Emad, Mhammed Alhassan Musallam, Isra Z. M. Eltazi, Zulfa Omer, Hiba Malik, Mayada O. E. Mohamed, Ali A. Elhassan, Eman O. E. Mohamed, Ahmed K. M. A. Ahmed, Elhami A. A. Ahmed, Esraa Eltaraifee, Bidour K. Hussein, Amal S. I. Abd Allah, Lina Salah, Mohamed Nimir, Omnia M. Tag Elseed, Tasneem E. A. Elhassan, Abubakr Elbashier, Esraa S. A. Alfadul, Moneeb Fadul, Khalil F. Ali, Shaimaa Omer M. A. Taha, Elfatih E. Bushara, Mutaz Amin, Mahmoud Koko, Muntaser E. Ibrahim, Ammar E. Ahmed, Liena E. O. Elsayed, Giovanni Stevanin

**Affiliations:** 1 Faculty of Medicine, University of Khartoum, Khartoum, Sudan; 2 Paris Brain Institute - ICM, CNRS UMR7225, INSERM 1127, Sorbonne University, F-75000 Paris, France; 3 Center of Neurodevelopmental Disorders (KIND), Centre for Psychiatry Research, Department of Women’s and Children’s Health, Karolinska Institutet and Stockholm Health Care Services, Region Stockholm, Stockholm, Sweden; 4 Astrid Lindgren Children’s Hospital, Karolinska University Hospital, Solna, Sweden; 5 Division of Pediatric Neurology, Department of Pediatrics, College of Medicine, King Saud University, Riyadh, Saudi Arabia; 6 College of Medicine, AlMughtaribeen University, Khartoum, Sudan; 7 Division of Neurology, Sudan Medical Council, Khartoum, Sudan; 8 Pediatric Neurology Department, Red Cross Memorial Children Hospital (RCWMCH), University of Cape Town (UCT), Cape Town, South Africa; 9 Faculty of Medicine, Omdurman Islamic University, Khartoum, Sudan; 10 Sudanese Neurogenetics Research group, Faculty of Medicine, University of Khartoum, Khartoum, Sudan; 11 National University Biomedical Research Institute, National University, Khartoum, Sudan; 12 Neurology Department, Hamad Medical Corporation, Doha, Qatar; 13 Department of Hematology and Medical Oncology, University of Cincinnati Medical Center, Ohio, USA; 14 Department of Neurology, Cambridge University Hospitals NHS Foundation Trust, Cambridge, CB2 0QQ UK; 15 Division of Emergency Medicine, Sudan Medical Specialization Board, Khartoum, Sudan; 16 Sudan Neuroscience Projects, University of Khartoum, Khartoum, Sudan; 17 Department of Molecular Neuroscience, Graduate school of Medicine, Osaka University, 2-2, Yamadaoka, Suita, Osaka 565-0871 Japan; 18 WPI Immunology Frontier Research Center, Osaka University, 3-1, Yamadaoka, Suita, Osaka 565-0871 Japan; 19 Institute of Endemic Diseases, University of Khartoum, Khartoum, Sudan; 20 Department of Pathology, University Hospitals Coventry and Warwickshire NHS Trust, Coventry, UK; 21 Warwick Medical School, University of Warwick, Coventry, UK; 22 Institute of Immunology and Immunotherapy, University of Birmingham, Birmingham, UK; 23 Department of Cardiology, Royal Derby Hospital, Derby, UK; 24 Department of Radiology, Universal Hospital, Khartoum, Sudan; 25 Department of Biochemistry and Molecular Biology, Faculty of Medicine, Al-Neelain University, Khartoum, Sudan; 26 Department of Basic Sciences, College of Medicine, Princess Nourah bint Abdulrahman University, Riyadh, P.O.Box 84428, Riyadh, 11671 Saudi Arabia; 27 Univ. Bordeaux, CNRS, INCIA, UMR 5287, F-33000 Bordeaux, France; 28 EPHE, PSL Research university, CNRS, INCIA, UMR 5287, F-75000 Paris, France

**Keywords:** Genetics research, Disease genetics

## Abstract

Hereditary spinocerebellar degenerations (SCDs) is an umbrella term that covers a group of monogenic conditions that share common pathogenic mechanisms and include hereditary spastic paraplegia (HSP), cerebellar ataxia, and spinocerebellar ataxia. They are often complicated with axonal neuropathy and/or intellectual impairment and overlap with many neurological conditions, including neurodevelopmental disorders. More than 200 genes and loci inherited through all modes of Mendelian inheritance are known. Autosomal recessive inheritance predominates in consanguineous communities; however, autosomal dominant and X-linked inheritance can also occur. Sudan is inhabited by genetically diverse populations, yet it has high consanguinity rates. We used next-generation sequencing, genotyping, bioinformatics analysis, and candidate gene approaches to study 90 affected patients from 38 unrelated Sudanese families segregating multiple forms of SCDs. The age-at-onset in our cohort ranged from birth to 35 years; however, most patients manifested childhood-onset diseases (the mean and median ages at onset were 7.5 and 3 years, respectively). We reached the genetic diagnosis in 63% and possibly up to 73% of the studied families when considering variants of unknown significance. Combining the present data with our previous analysis of 25 Sudanese HSP families, the success rate reached 52–59% (31–35/59 families). In this article we report candidate variants in genes previously known to be associated with SCDs or other phenotypically related monogenic disorders. We also highlight the genetic and clinical heterogeneity of SCDs in Sudan, as we did not identify a major causative gene in our cohort, and the potential for discovering novel SCD genes in this population.

## Introduction

Hereditary forms of spastic paraplegia (HSP), cerebellar ataxia, and spinocerebellar ataxia are distinct but overlapping clinical entities caused by related mechanisms that encompass a continuum of phenotypes [1–3]. We refer hereafter to these disorders as hereditary spinocerebellar degenerations (SCDs).

SCDs are characterized clinically by ataxia and/or spasticity complicated, in some cases, by other neurological or extra-neurological manifestations [1, 2]. They have more than 220 subtypes that afflict ~1:10,000 individuals worldwide with evident phenotypic and genetic heterogeneity and clinical overlap with other neurogenetic conditions such as intellectual disabilities, motor neuron diseases, encephalopathies, or neurodevelopmental disorders [4, 5]. The advent of next-generation sequencing (NGS) has markedly boosted SCDs diagnosis in recent years by the identification of a multitude of causative mutations in a large variety of disease-causing genes [4]. Dysfunction of mitochondria, ion channels, and cellular metabolism are the main altered functions by the pathogenic variants in these genes in addition to the abnormal expansions of nucleotide repeats [4].

Sudan is an East-African country with complex genetic and population structures [6]. This complexity stemmed from the linguistic and cultural differences between its ethnic groups acting in parallel with other, sometimes opposing, population genetic forces, e.g., consanguinity, admixture, and migration [6–9]. For instance, 67% of marriages in some parts of the country are consanguineous (42% first-degree cousins and 25% fifth-degree consanguinity and above) [10].

In a previous study, we screened 25 Sudanese families with HSP for mutations in 68 HSP genes using NGS targeted gene panel and reached a genetic diagnosis in 28% of these families [11]. In the current study, we investigated 38 novel Sudanese families with SCDs using a combination of candidate gene approaches, NGS targeted gene panel screening, and whole-exome sequencing (WES). We documented the studied patients’ clinical presentations and compared the diagnostic utility of the approaches in the two studies.

## Subjects and Methods

### Patients recruitment and interviews

We included a total of 90 patients from 38 Sudanese families in this study with the following inclusion criteria:

1. Patients presenting with symptoms, signs, and/or history suggestive of SCD.
2. Non-genetic causes that can mimic neurological illnesses that resemble SCD due to pregnancy- or birth-related insults, as well as toxic exposures, have been excluded through interview of the family members. MRI, when available, excluded tumor or compressions of CNS structures.
3. Participants from the family (patients and at least two healthy subjects) or their guardian (in the case of participants below 18 years old or patients with intellectual disabilities), agreed to participate in the study. We also examined and samples healthy subjects such as the parents, siblings, second-degree relatives, and/or third-degree relatives, in priority order depending on their availability, in order to help in the variant filtering. They had to be older than patients’ age at disease onset.
4. Sudanese by descent.
5. Presence of multiple cases affected in the same family, or sporadic case from a consanguineous marriage, to increase the probability to identify genetic causes.

Four out of the 38 families were screened in our previous study without reaching a genetic diagnosis [11]. Index cases were recruited from multiple neurology and pediatrics neurology clinics in Khartoum, the capital of Sudan that gather most tertiary hospitals and specialized clinics in the country. Most families originated from outside the city. Patients and families were interviewed and examined at the Department of Biochemistry, Faculty of Medicine, University of Khartoum, Sudan; the Pediatric Neurology Clinics, Soba University Hospital, Sudan; or the families’ residences in the capital of Sudan, Khartoum, or other Sudanese cities. The diagnosis protocol followed the EUROSPA/SPATAX clinical criteria (https://spatax.wordpress.com/downloads/). We collected 2 ml of saliva from the patients and healthy-related subjects using Oragene®•DNA (OG-500 and OG-575) kits (DNA Genotek Inc., Ottawa, ON, Canada).

### The strategy of genetic studies

**Fig. 1:**
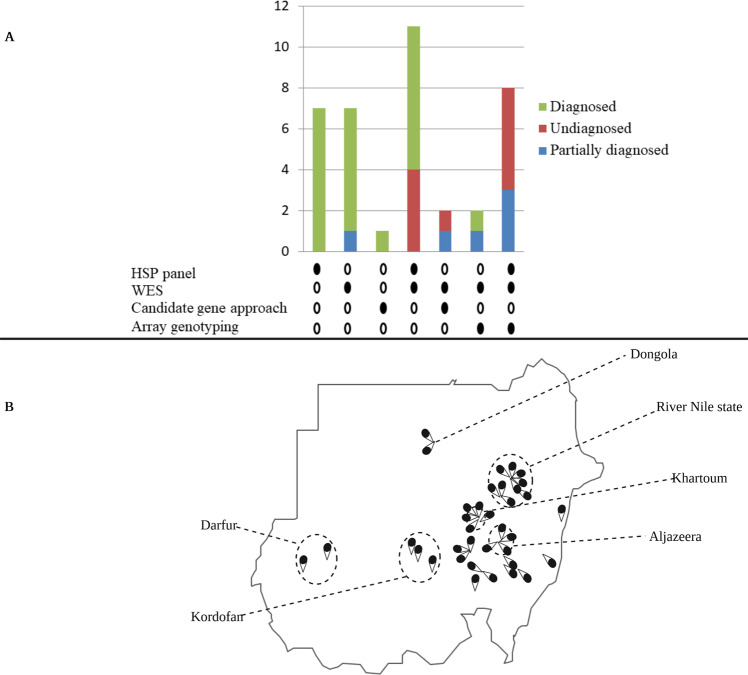
Genetic tools and geographical origins. Genetic tools used for investigating our families and their utility (**A**) and the geographical origin of the families (**B**). **A** More than one genetic diagnostic approach was used in 23 out of 38 families, including all the undiagnosed families. Twenty-six families were investigated initially using HSP-targeted NGS gene panel (HSP panel) screening. Of these, eleven families were further investigated using WES and eight using WES and array genotyping. Eleven other families were directly investigated using WES, without HSP panel screening. Candidate gene approach was used in three families, two that were screened for repeat expansion-associated autosomal dominant spinocerebellar ataxias, and one for Friedreich’s ataxia repeat expansion. Sanger sequencing (not shown) was used for testing the segregation of all the identified candidate variants, except repeats expansion variants. HSP panel, hereditary spastic paraplegia next-generation sequencing targeted gene panel; WES, whole-exome sequencing. The families partially diagnosed relate to families where only a fraction of the patients was diagnosed. The numbers on the y-axis indicate the number of families; the filled circles indicate the used genetic tool. **B** The figure shows regions, states, or cities in Sudan from which the studied families originated (each pin-drop represents a single family).

In this study, most families were studied using more than one diagnostic modality. We used various combinations of different genetic approaches, including NGS targeted gene panel screening, WES, candidate gene approach, and array genotyping (Fig. 1A). Initially, our experimental design was to screen the patients for mutations in selected genes depending on their phenotypes and to subsequently perform WES in negative cases. Later, with the drop in the costs of WES, we skipped the screening steps, except in patients with suspected repeat expansions based on clinical and inheritance data. Array genotyping was mainly used for homozygosity mapping and detection of copy number variations (CNVs). The presence of CNVs was also tested through coverage analysis in NGS data (gene panel and WES). Twenty-six families were investigated initially using HSP-targeted NGS gene panel (HSP panel) screening. Of these, eleven families were further investigated using WES and eight using WES and array genotyping. Eleven other families were directly investigated using WES, without HSP panel screening. Candidate gene approach was used in three families, two that were screened for repeat expansion-associated autosomal dominant spinocerebellar ataxias, and one for Friedreich’s ataxia repeat expansion.

### DNA extraction and quality check

We extracted DNA from saliva following the prepIT®.L2P manual protocol provided by the manufacturer (DNA Genotek). DNA quantity (Absorbance at 260 nm) and quality (check of the high molecular weight DNA, absorbance ratio 260/280 and 260/320) were checked using a NanoDrop spectrophotometer (Thermo Scientific, Wilmington, DE, USA), a Qubit® fluorometer (Promega, Madison, WI, USA), and standard agarose gel electrophoresis.

### Next-generation panel screening of HSP genes

Fifty µl of patients’ DNA solution at a concentration of 50 ng per dl were sent for NGS panel screening at the genotyping and sequencing core facility of the Paris Brain Institute - ICM, Paris, France. A double capture enrichment strategy was used (Roche NimbleGen® SeqCap® Ez, USA). Sequencing was done on the MiSeq® platform (Illumina, CA, USA). Detailed methodology and bioinformatics analysis are available in previous reports [11, 12]. We systematically searched for point variations and genomic rearrangements. We targeted a median depth coverage > 100, and variants affecting positions with coverage < 30 were reanalyzed by Sanger sequencing if located in convincing causative genes based on the clinical presentation and inheritance mode.

### Whole-exome sequencing

Twenty µl of DNA solution at a concentration of 20 ng per dl were sent for WES at the genotyping and sequencing core facility of the Paris Brain Institute - ICM, Paris, France. Exons were captured on the genomic DNA using the SeqCap® EZ MedExome Kit (Roche, IN, USA), followed by massively parallel sequencing on a Novaseq® 6000 sequencer (Illumina, CA, USA). Except for aligning reads to the hg37 version of the human genome (NCBI) using Burrows-Wheeler Aligner software, we processed exome data up to the calling of variants using the Genome AnalysisToolkit software (GATK) following the GATK4 best-practice pipeline.

We annotated and prioritized variants using software included in VarAFT annotation and filter tool [13]. Data analysis and variants filtration were carried out based on the minor allele frequency, the variant’s effect, and in silico prediction. We filtered all variants with allele frequencies < 0.0001 in the GnomAD genome database. First, we examined variants with predicted major structural effects; nonsense, stop loss, frameshift, and canonical splice site variants. After checking for loss of function variants, we examined missense variants annotated as pathogenic by Sift and Polyphen software [14, 15] and non-frame-shift variants. To verify that we had not missed strong candidate variants due to our conservative frequency filter, we repeated the analysis using a frequency cut-off of 0.001 in the GnomAD genome database. In this study, we focused the analysis to Online Inheritance in Man (OMIM) disease-related genes (https://www.omim.org/) and recently published ataxia or HSP-causative genes with strong evidence from the literature. When multiple affected relatives were processed from the same family, they were analyzed together according to the suspected inheritance mode and then individually to take into account possible phenocopies. Genomic rearrangements were tested using PennCNV-1.0.5 [16].

### Sanger sequencing

Primers were designed using Primer3 Plus software [17]. DNA was amplified on a GeneAmp^®^ PCR System 9700 (Thermo Fisher, MA, USA). We checked the quantity and quality of PCR products, including product size and off-target amplification, using the Caliper^®^LabChip GX System and its related software (PerkinElmer, MA, USA) according to the manufacturer’s protocol. Sanger sequencing was then done at the labs of Eurofins Genomics (Germany) using the Big Dye Chemistry in an ABI3730 automated sequencer (Applied Biosystems, Thermo Fisher Scientific, USA) using the procedures recommended by the manufacturer on the PCR product. Sequencing files (ABI format) were then visualized and analyzed using Sequence Scanner Software^®^ v2.0 (Thermo Fisher Scientific, USA).

### Array genotyping

Two hundred nanograms of genomic DNA from participating members of the families F5, F41, F54, F65, F70, F73, F74, F75, F80, F81, and F85 were sent for genotyping at the Pitié-Salpêtrière Post-Genomic Platform (P3S), Paris, France. Genotyping was performed on Illumina Infinium OmniExpress-24vl-3-A1 array, which contained ~ 710,000 SNP markers. Raw data were analyzed at the P3S platform using GenomeStudio™ Software. Runs of homozygosity were performed using version 1.07 of Plink software [18] to prioritize the variants in WES analysis. Candidate pathogenic copy number variants (CNV) were searched using PennCNV-1.0.5 software [16].

### Repeats expansion detection

Genomic DNA from patients with clinical presentations and pedigree structures suggestive of dominant spinocerebellar ataxias (F49 and F65) or Friedreich’s ataxia (F38) were screened for repeats expansion using specific PCR-based approaches at the genetics departments of the Pitié-Salpêtrière Hospital and University Hospital of Montpellier, France, respectively. From the dominant spinocerebellar ataxias, we screened for pathogenic DNA repeat expansions in the SCA genes *ATXN1* (SCA1), *ATXN2* (SCA2), *ATXN3* (SCA3), *CACNA1A* (SCA6), *ATXN7* (SCA7), *TBP* (SCA17), and *ATN1* (DRPLA) using a multiplex PCR amplification followed by capillary electrophoresis in a 3730 ABI sequencer (Applied Biosystems). The *FRDA* gene-associated repeat was amplified by a repeat-primed PCR approach.

## Results

**Fig. 2:**
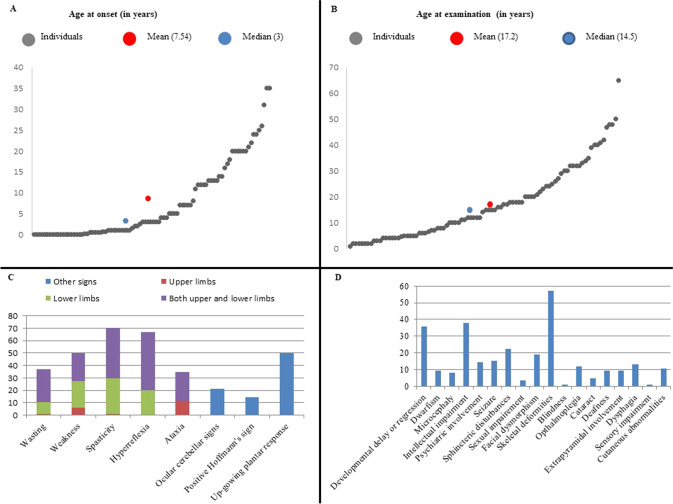
Clinical overview of the cohort. **A** Patients’ age-at-examination. The mean age-at-examination was 17.2 years. **B** Age-at-onset of the SCDs in our patients. The mean age-at-onset was 7.54 years. **C** Signs detected during patients’ examination. The percentages of patients with pyramidal and cerebellar signs are shown. The majority of our patients presented with pyramidal features. **D** Features complicating the SCDs phenotype in our cohort. Skeletal deformities, intellectual impairment, and developmental delay and/or regression are the most common features complicating the SCDs phenotype in our cohort.

We studied 38 families (90 sampled affected patients), each including at least one patient manifesting features of SCDs. The studied families originated from multiple regions in Sudan, though the distribution is markedly skewed towards the central parts of the country. More than one-fifth of the families (23.6%) originated from a single state in central Sudan, the River Nile state (Fig. 1B). The number of affected males and females in our cohort was approximately equal (53% males vs. 47% females). However, the patients’ age at examination distribution was less homogenous; most patients were less than 18 years old. The mean and median patients’ ages-at-examination were 17.24 (SD = 13.97) and 14.5 years, respectively (Fig. 2B).

### Disease phenotypes

Most patients had an early-onset disease; the mean and median ages-at-onset were 7.44 (SD = 9.11) and three years, respectively (Fig. 2A). Most of the patients in our cohort had spasticity (70%). Limb ataxia was noted only in ~30% of the patients, while ocular cerebellar signs were noted in 20% (Fig. 2C). A pure SCDs phenotype was noted in ~14% of the patients, nine presented with a pure HSP while four presented with pure cerebellar ataxia. The most common features complicating the SCD phenotype in our patients were skeletal deformities, developmental delay or regression, and intellectual impairment (Fig. 2D). Table 1 summarizes the clinical presentation and the genetic diagnosis in each family where appropriate, and detailed phenotype of all the patients in our cohort are available per patient in the Supplementary Table. The diversity of clinical association did not allow us to distinguish a frequent phenotype that could have been analyzed separately as a whole. However, several families presented with similar clinical presentations, such as families F63 and F84, but they finally appeared to segregate mutations in different genes.

**Table 1:** Overview of the clinical presentation of the families in our cohort with the OMIM corresponding identity deduced from the genetic results.

Family code	Clinical features observed in patients	OMIM phenotype (MIM ID)	Gene
F8	Cerebellar ataxia, cataract, and hypotonia	Marinesco-Sjogren syndrome (# 248800)	*SIL1*
F27	Stunted growth, dysmorphic features, microcephaly, cerebellar ataxia, and retinitis pigmentosum	Cockayne syndrome A (# 216400)	*ERRC8*
F31	Cerebellar ataxia and developmental delay	Cerebellar ataxia, mental retardation, and disequilibrium syndrome 1 (# 224050)	*VLDLR*
F38	Cerebellar ataxia	Friedreich ataxia (# 229300)	*FXN*
F41	Cafe-au-lait spots, cerebellar ataxia, and skeletal deformities	Neurofibromatosis, type 1 (# 162200)	*NF1*
F49	Cerebellar ataxia, spasticity, and dysarthria	Machado-Joseph disease (# 109150)	*ATXN3*
F53	Spastic limbs, wasting, and low-set ears	Spastic paraplegia 11, autosomal recessive (# 604360)	*SPG11*
F54	Developmental delay and spastic limbs	Spastic paraplegia 48, autosomal recessive (# 613647)	*AP5Z1*
F57	Spastic limbs, skeletal deformities, cerebellar ataxia, wasting, and weakness	Spastic ataxia, Charlevoix-Saguenay type (# 270550)	*SACS*
F59	Intellectual disability, spastic limbs, and skeletal deformities	Spastic paraplegia 45, autosomal recessive (# 613162)	*NT5C2*
F61	Regressed developmental milestones, spastic limbs, cerebellar ataxia, and ophthalmoplegia	Spastic paraplegia 35, autosomal recessive (# 612319)	*FA2H*
F62	Stunted growth, intellectual disability, spastic limbs, dysarthria, wasting, and cerebellar ataxia	Cockayne syndrome B (# 133540)	*ERCC6*
F63	Intellectual disability, aggression, dysmorphic features, squint, skeletal deformities, and spastic limbs (ref. 19)	Mental retardation, autosomal recessive 36 (# 615286)	*ADAT3*
F66	Developmental delay, spastic limbs, and weakness	Novel phenotype in a known gene not associated previously with SCD	*DMXL2 (VUS)*
F67	Cerebellar ataxia, epilepsy, and learning disability	Ceroid lipofuscinosis, neuronal, 7 (# 610951)	*MFSD8 (VUS)*
F68	Abnormal gait, spasticity, and cerebellar ataxia	Spastic paraplegia 35, autosomal recessive (# 612319)	*FA2H*
F70	Weakness, wasting, dysphagia, developmental delay/regression, and unsteady gait	Leukodystrophy, hypomyelinating, 7, with or without oligodontia and/or hypogonadotropic hypogonadism (# 607694)	*POLR3A*
F76	Developmental delay, spasticity, wasting, and skeletal deformities	Spastic paraplegia 54, autosomal recessive (# 615033)	*DDHD2*
F78	Spasticity, cerebellar ataxia, weakness, intellectual disability, and ears of the lynx sign on MRI	Spastic paraplegia 15, autosomal recessive (# 270700)	*ZFYVE26*
F79	Developmental delay, dysmorphic features, abnormal gait, autistic features, and behavioral disturbances (ref. 19)	Mental retardation, autosomal recessive 38 (# 615516); spinocerebellar ataxia, X-linked 1 (# 302500)	*HERC2/ ATP2B3 (VUS)*
F80	Developmental delay, spastic limbs, and skeletal deformities	Spastic paraplegia 45, autosomal recessive (# 613162)	*NT5C2*
F81	Developmental delay, spastic limbs, and epilepsy (ref. 19)	Novel phenotype in a known gene not associated previously with SCD.	*CCDC82*
F82	Global developmental delay, microcephaly, dysmorphic features, epilepsy, and spastic lower limbs	Mental retardation, X-linked, syndromic, turner type (# 309590)	*HUWE1*
F83	Early-onset pure hereditary spastic paraplegia (ref. 19)	Spinocerebellar ataxia 40 (# 616053)	*CCDC88C*
F84	Developmental delay/regression, microcephaly, squint, and generalized spasticity	Mucolipidosis IV (# 252650)	*MCOLN1*
F85	Deafness and mutism, mild cerebellar ataxia, spasticity	Deafness, autosomal recessive 84a (# 613391)	*PTPRQ (VUS)*
FM3	Global developmental delay, spastic limbs, and skeletal deformities (ref. 27)	Neurodevelopmental disorder with microcephaly, hypotonia, and variable brain anomalies (# 617481)	*PRUNE1*
FM6	Spasticity and ocular cerebellar signs (ref. 39)	Leukoencephalopathy with brainstem and spinal cord involvement and lactate elevation (# 611105)	*DARS2*
**The undiagnosed families**			
F5	Spasticity and cerebellar ataxia	Not applicable	
F46	Skeletal deformities and cerebellar ataxia	Not applicable	
F74	Weakness, myopathic faces, contractures, and hypophonia	Not applicable	
**Families with mutations in candidate novel genes**			
F7	Pure hereditary spastic paraplegia	Unpublished	
FM2	Cerebellar ataxia, spasticity, epilepsy, dysphagia, extrapyramidal features, and brain MRI white matter abnormalities.	Unpublished	
F65	Late-onset ataxia and spasticity	Unpublished	
F69	Febrile convulsions, global developmental delay, spastic limbs, and skeletal deformities (ref. 30)	Spastic Paraplegia 86, autosomal recessive (# 619735)	*ABHD16A*
F73	Slurred speech, gait abnormalities, and epilepsy	Unpublished	
F75	Weakness and spasticity	Unpublished	
F77	Global developmental delay, spasticity, and convulsions	Unpublished	

*OMIM* Online inheritance in man. More detailed clinical information is provided in the supplementary table.

### Genetic tests results

When focusing the analysis on known genes involved in neurogenetic conditions, we reached a genetic diagnosis in 63% (24/38) of the studied families, possibly 73% (28/38) if including families with variants of uncertain significance (VUS). In most of these families (23/28, 82%), diagnosis concerned all patients of the family that could be tested. The candidate variants in the families F41, F85, F54, F70, and F80 were not identified in all the patients within the family (Table 2 and the Supplementary material). This partial segregation was probably due to the high consanguinity rate that concentrated several disease-causing mutations or non-genetic phenocopies in the same family.

**Table 2:** Overview of the genetic data in our patients (full cohort).

Family code	Patient ID	Consanguinity	Gender	Genetic tests performed	Gene (causative variant in known SCD genes)	Gene (VUS or CN)
F5	53	yes	M	WES, array genotyping	none	none
F5	56	yes	M	Array genotyping	none	none
F5	61	yes	M	WES, array genotyping	none	none
F7	85	yes	M	WES, Sanger seq. validation	none	CN gene
F7	86	yes	F	WES, Sanger seq. Validation	none	CN gene
F8	98	yes	F	WES, Sanger seq. validation	*SIL1*	none
F8	99	yes	F	WES, Sanger seq. validation	*SIL1*	none
F27	227	no	M	WES, Sanger seq. validation	*ERCC8*	none
F27	228	no	F	WES, Sanger seq. validation	*ERCC8*	none
F27	229	no	F	WES, Sanger seq. validation	*ERCC8*	none
F31	267	yes	F	WES, Sanger seq. validation	*VLDLR*	none
F31	268	yes	F	WES, Sanger seq. validation	*VLDLR*	none
F38	318	yes	M	WES; FRDA screening	*FRDA* expansion	none
F38	317	yes	M	Sanger seq. validation	*FRDA* not tested	none
F38	319	yes	F	Sanger seq. validation	*FRDA* not tested	none
F38	328	yes	M	not sampled	not sampled	not sampled
F38	329	yes	F	WES	*FRDA* not tested	none
F41	351	yes	F	WES, array genotyping, Sanger seq. validation	*NF1*	none
F41	349	yes	M	WES, array genotyping, Sanger seq. validation	no segreg of *NF1*	none
F46	380	yes	M	WES	none	none
F46	383	yes	F	Not sampled	Not sampled	Not sampled
F49	396	no	M	SCA expansion screening	*SCA3*	none
F49	398	no	M	SCA expansion screening	*SCA3*	none
FM2	2016	yes	F	WES, Sanger seq. validation	none	CN gene
FM2	2008	yes	M	Sanger seq. validation	none	no segreg of CN gene
FM2	2013	yes	F	WES, Sanger seq. validation	none	CN gene
FM3	2020	yes	M	WES, microsatelittes genotyping, Sanger seq. validation	*PRUNE1*	none
FM3	2021	yes	F	Microsatelittes genotyping, Sanger seq. validation	*PRUNE1*	none
FM3	2022	yes	F	WES, microsatelittes genotyping, Sanger seq. validation	*PRUNE1*	none
FM6	2042	yes	F	Sanger seq. validation	*DARS2*	none
FM6	2043	yes	F	WES, Sanger seq. validation	*DARS2*	none
FM6	2044	yes	F	Sanger seq. validation	*DARS2*	none
F53	417	no	M	Sanger seq. validation	*SPG11*	none
F53	418	no	M	Sanger seq. validation	*SPG11*	none
F53	419	no	M	HSP panel screening, Sanger seq. validation	*SPG11*	none
F54	427	yes	M	HSP panel, WES, array genotyping, Sanger seq. validation	*AP5Z1*	none
F54	426	yes	F	WES, array genotyping, Sanger seq. validation	no segreg of *AP5Z1*	none
F57	439	yes	F	Sanger seq. validation	*SACS*	none
F57	440	yes	M	Sanger seq. validation	*SACS*	none
F57	441	yes	F	HSP panel, Sanger seq. validation	*SACS*	none
F59	451	yes	F	Sanger seq. validation	*NT5C2*	none
F59	452	yes	M	HSP panel, Sanger seq. validation	*NT5C2*	none
F59	453	yes	M	Sanger seq. validation	*NT5C2*	none
F61	465	yes	M	HSP panel, Sanger seq. validation	*FA2H*	none
F61	467	yes	F	Sanger seq. validation	*FA2H*	none
F62	470	yes	M	WES, Sanger seq. validation	*ERCC6*	none
F62	471	yes	M	HSP panel, WES, Sanger seq. validation	*ERCC6*	none
F63	476	yes	F	HSP panel, WES, Sanger seq. validation	*ADAT3A*	none
F63	477	yes	F	WES, Sanger seq. validation	*ADAT3A*	none
F65	484	yes	M	Array genotyping, Sanger seq. validation	none	CN gene
F65	485	yes	M	SCA expansion screening, WES, array genotyping, Sanger seq. validation	none	CN gene
F65	486	yes	M	HSP panel, SCA expansion screening, WES, array genotyping, Sanger seq. validation	none	CN gene
F65	487	yes	F	Array genotyping, Sanger seq. validation	none	CN gene
F66	490	yes	M	HSP panel, WES, Sanger seq. validation	none	*DMXL2*
F66	493	yes	M	HSP panel, WES, Sanger seq. validation	none	*DMXL2*
F67	496	yes	F	WES, Sanger seq. validation	none	*MFSD8*
F68	503	yes	F	HSP panel, WES, Sanger seq. validation	*FA2H*	none
F68	504	yes	F	HSP panel, WES, Sanger seq. validation	*FA2H*	none
F69	508	yes	F	HSP panel, WES, Sanger seq. validation	none	CN gene *(ABHD16A)*
F69	509	yes	M	HSP panel, WES, Sanger seq. validation	none	CN gene *(ABHD16A)*
F70	513	yes	F	WES, array genotyping, Sanger seq. validation	*POLR3A*	none
F70	514	yes	M	HSP panel, WES, Sanger seq. Validation, array genotyping	no segreg of *POLR3A*	none
F73	527	yes	M	HSP panel, WES, Sanger seq. Validation, array genotyping	none	CN gene
F73	525	yes	F	HSP panel, WES, Sanger seq. Validation, array genotyping	none	no segreg of CN gene
F74	529	yes	M	Array genotyping, Sanger seq. validation	none	none
F74	530	yes	M	HSP panel, WES, Sanger seq. Validation, array genotyping	none	none
F74	531	yes	M	HSP panel, WES, Sanger seq. Validation, array genotyping	none	none
F74	532	yes	M	Array genotyping, Sanger seq. validation	none	none
F74	533	yes	M	Array genotyping, Sanger seq. validation	none	none
F74	535	yes	M	Array genotyping, Sanger seq. validation	none	none
F74	536	yes	M	Array genotyping, Sanger seq. validation	none	none
F75	542	yes	F	HSP panel, WES, Sanger seq. Validation, array genotyping	none	CN gene
F75	543	yes	M	HSP panel, WES, Sanger seq. Validation, array genotyping	none	CN gene
F76	547	yes	F	HSP panel, Sanger seq. validation	*DDHD2*	none
F76	548	yes	M	HSP panel, Sanger seq. validation	*DDHD2*	none
F77	550	yes	F	HSP panel, WES, Sanger seq. validation	none	CN gene
F77	551	yes	M	HSP panel, WES, Sanger seq. validation	none	CN gene
F78	557	yes	M	HSP panel, Sanger seq. validation	*ZFYVE26*	none
F78	AA	yes	F	Sanger seq. validation	*ZFYVE26*	none
F79	568	yes	M	WES, Sanger seq. validation	*none*	*HERC2/ATP2B3*
F80	573	yes	F	WES, array genotyping, Sanger seq. validation	*NT5C2*	none
F80	572	yes	F	WES, array genotyping, Sanger seq. validation	no segreg of *NT5C2*	none
F81	576	yes	M	WES, array genotyping, Sanger seq. validation	*CCDC82*	none
F81	577	yes	F	WES, array genotyping, Sanger seq. validation	*CCDC82*	none
F82	580	yes	M	WES, Sanger seq. validation	*HUWE1*	none
F83	581	distant	F	WES, Sanger seq. validation	*CCDC88C*	none
F84	588	yes	M	WES, Sanger seq. validation	*MCOLN1*	none
F84	589	yes	F	WES, Sanger seq. validation	*MCOLN1*	none
F85	2056	yes	M	WES, array genotyping, Sanger seq. validation	none	*PTPRQ* homozygous
F85	2059	yes	F	Array genotyping, Sanger seq. validation	none	*PTPRQ* heterozygous

*M* Males, *F* Females, *CN gene* Candidate novel gene, *WES* Whole exome sequencing, *FRDA* Friedreich ataxia gene (GAA expansion), *SCA3* Repeat expansion in the ATXN3 gene, *HSP panel* Hereditary spastic paraplegia gene panel, *no cosegreg* no cosegregation of the variant (absent in the patient)

### Inheritance patterns

**Fig. 3:**
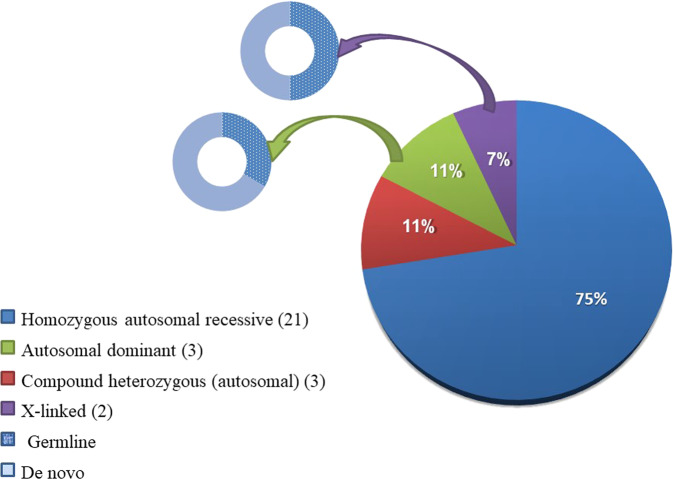
The pattern of inheritance in the families with mutations in known disease genes. Compound heterozygous inheritance is separated from homozygous autosomal recessive inheritance to highlight the effect of consanguinity. One family, F79, was counted in two inheritance modes as it segregated two likely causative variants with different patterns of inheritance but both possibly contributing to the phenotype as we reported previously (Ref. 19).

The pattern of inheritance in most of the possibly diagnosed families was an autosomal recessive pattern in 24 of them (Fig. 3); of note, F79 was counted in two inheritance modes as it segregated two likely causative variants with different patterns of inheritance but both possibly contributing to the phenotype as we reported previously [19]). Most autosomal recessive families were segregating homozygous variants (75%), while compound heterozygous variants were observed in 11%. Autosomal dominant inheritance was identified in 11% of the families, and two families showed X-linked inheritance (Fig. 3).

### Genetic variants

We identified 31 different variants in known disease genes in this study (Table 3): 26 causative or likely causative variants in 24 families and five variants of unknown significance (VUS) in four families. One variant in *FA2H*, NM_024306.5:c.674 T > C (p.Leu225Pro), was identified twice in families F61 and F68 who shared a related phenotype. Most of the variants we identified were missense variants (12/31, 39%), followed in frequency by splice-site (23%) and frameshift (19%) variants. We identified pathogenic repeat expansions in two families and nonsense variants in three families. Array genotyping didn’t detect candidate CNV or chromosomal rearrangements. Each variant was validated by VariantValidator version 2.1.1 and classified by the authors after evaluating the entire clinical situation (Supplementary table), segregation analysis (Supplementary material) and the ACMG 2015 classification (Table 3).

**Table 3:** Causative variants and variants of uncertain significance (VUS) identified in genes previously known to be associated with neurological phenotypes.

Family code	Inheritance pattern	Gene	Variant	ACMG class for point mutations / Conclusion from other criteria	ACMG criteria
**Families in which the candidate variant explains the disease in all the patients**					
F8	AR homozygous	*SIL1*	NM_001037633.2:c.767 + 1 G > A (splice-site)	Pathogenic	PVS1, PM2, PP1, PP4
F27	AR compound heterozygous	*ERCC8*	NM_000082.4:c.523 T > C (p.Ser175Pro) / NM_000082.4:c.572_574del (p.Ala191del)	Likely pathogenic/likely pathogenic	PM2, PP1, PP3, PP4 / PM2, PP1, PP3, PP4, BP3
F31	AR homozygous	*VLDLR*	NM_003383.5:c.2144 G > A (p.Cys715Tyr)	Likely pathogenic	PP1 (moderate), PM2, PP3, PP4
F38	AR homozygous	*FXN*	GAA repeat expansion (a single patient tested but phenotype compatible in other patients)	Not applicable/Toxic expansion	
F49	AD	*ATXN3*	CAG repeat expansion (73 + /−3 CAG)	Not applicable/Toxic expansion	_
F53	AR compound heterozygous	*SPG11*	NM_025137.4:c.2399del (p.Tyr800Phefs*19) / NM_025137.4:c.6739_6742del (p.Glu2247Leufs*14)	Pathogenic/pathogenic	PVS1, PM2, PM3, PP1/ PVS1, PM2, PP1, PP5
F57	AR homozygous	*SACS*	NM_014363.6:c.10444_10447del (p.Leu3482Glnfs*12)	Pathogenic	PVS1, PM2, PP1
F59	AR homozygous	*NT5C2*	NM_001351171.2:c.175 + 1 G > A (splice-site)	Pathogenic	PVS1, PM2, PP1, PP5
F61	AR homozygous	*FA2H*	NM_024306.5:c.674 T > C (p.Leu225Pro)	Likely pathogenic	PP1 (strong), PM2, PP3, PP4
F62	AR homozygous	*ERCC6*	NM_000124.4:c.4063–1 G > C (splice-site)	Pathogenic	PVS1, PM2, PP1, PP4, PP5
F63	AR homozygous	*ADAT3*	NM_138422.4:c.430 G > A (p.Val144Met)	Pathogenic	PM2, PP1 (strong), PS3, PP3, PP4, PP5
F66	AR homozygous	*DMXL2*	NM_001174116.3:c.5020 A > C (p.Lys1674Gln)	VUS	PM2, PP1, PP3, BP1
F67	AR homozygous	*MFSD8*	NM_152778.3:c.753 A > G (p.Glu251Glu) (splice site)	VUS	PM2
F68	AR homozygous	*FA2H*	NM_024306.5:c.674 T > C (p.Leu225Pro)	Likely pathogenic	PP1 (strong), PM2, PP3, PP4
F76	AR homozygous	*DDHD2*	NM_015214.3:c.985 C > T (p.Arg329*)	Pathogenic	PVS1, PM2, PP5, PP1, PP3
F78	AR homozygous	*ZFYVE26*	NM_015346.4:c.1254dup (p.Cys419Valfs*61)	Pathogenic	PVS1, PM2, PP1, PP3
F79	AR homozygous / X-linked	*HERC2/ATP2B3*	NM_004667.6:c.10855 C > T (p.Pro3619Ser)/ NM_021949.3:c.2086 C > T (p.Arg696Cys)	VUS/VUS	PM2, PP3 / PM2, PP3
F81	AR homozygous	*CCDC82*	NM_001318736.2:c.535 C > T (p.Arg179*)	Pathogenic	PVS1, PM2, PP1
F82	X-linked (likely de novo)	*HUWE1*	NM_031407.7:c.12639 G > A (p.Met4213Ile)	Likely pathogenic	PM1, PM2
F83	AD (likely de novo)	*CCDC88C*	NM_001080414.4:c.1993G > A (p.Glu665Lys)	Likely pathogenic	PS3, PM2, PP3
F84	AR homozygous	*MCOLN1*	NM_020533.3:c.514 C > T (p.Arg172*)	Pathogenic	PVS1, PP5, PM2, PP1
FM3	AR homozygous	*PRUNE1*	NM_021222.3:c.132 + 2 T > C (splice-site)	Pathogenic	PVS1, PM2, PP1
FM6	AR compound heterozygous	*DARS2*	NM_018122.5:c.1762C > G (p.Leu588Val) / NM_018122.5:c.563 G > A (p.Arg188Gln)	Likely pathogenic/likely pathogenic	PM2, PP1 (moderate), PP3, PP4, PP5 / PM2, PP1 (moderate), PP3, PP4
**Families in which the candidate variant explains the disease only in a proportion of the patients**					
F41	AD (likely de novo)	*NF1*	NM_001042492.3:c.187_188del (p.Lys63Glufs*3)	Pathogenic	PVS1, PM2, PM6
F54	AR homozygous	*AP5Z1*	NM_014855.3:c.1132 G > A (p.Gly378Arg) (splice-site)	Likely pathogenic	PVS1, PM2
F70	AR homozygous	*POLR3A*	NM_007055.4:c.1771–7 C > G (splice-site)	Likely pathogenic	PP1 (strong), PS3, PM2, PP5
F80	AR homozygous	*NT5C2*	NM_001134373.3:c.629del (p.Tyr210Serfs*17)	Pathogenic	PVS1, PM2, PP3
F85	AR homozygous	*PTPRQ*	NM_001145026.2:c.5893 C > A (p.Pro1965Thr)	VUS	PM2, PP3

*AR* Autosomal recessive, *AD* Autosomal dominant, *PVS1* null variant in a gene where loss of function is a known disease mechanism, *PS3* well-established in vitro or in vivo functional studies supportive of a damaging effect on the gene or gene product, *PM1* located in a mutational hot spot or well-established critical protein domain without benign variation, *PM2* absent or have low frequency in gnomAD public database, *PM3* detected in trans with a pathogenic variant, *PM6* assumed de novo, *PP1* cosegregate with disease in multiple affected family members, *PP3* multiple lines of computational evidence support a deleterious effect, *PP4* Patients’ phenotype or family history is highly specific for a disease with a single genetic etiology, *PP5* reported as deleterious by a reputable source.

Approximately eighty percent of the candidate single nucleotide and insertion/deletion variants located in known disease genes (23/29, excluding the two nucleotide expansions) were either pathogenic or likely pathogenic, according to the ACMG 2015 guidelines for interpreting sequence variations [20].

Additionally, five likely causative variants fitted to the category of VUS but some with convincing evidence of pathogenicity, however. All the candidate deleterious VUS identified in this cohort segregated with the disease and could fit the categories of pathogenic or likely pathogenic variants if additional evidence is identified in the future. The VUS NM_152778.3:c.753 A > G (p.Glu251Glu), identified in family F67, is a synonymous variant but predicted to alter the splicing of *MFSD8* (TraP score 0.96; SpliceAI score 0.7) and cause skipping of exon 8. It was absent from the gnomAD v2.1.1 database and from 120 index cases of Sudanese origin with various neurological conditions. The patient presented with intellectual disability, cerebellar ataxia, and epilepsy (Supplementary Table), a phenotype suggestive of, but not exclusive to, neuronal ceroid lipofuscinosis. Furthermore, the patient had cousins who passed away in their early childhood after a similar illness. We considered it as a plausible candidate based on in-silico prediction tools and suggestive phenotype and family history.

The second VUS was in *DMXL2*, NM_001174116.3:c.5020 A > C (p.Lys1674Gln), and was identified in the two probands from family F66. It is predicted as pathogenic by Sift, Polyphen 2, MutationTaster [21], LRT [22], and Provean [23] and had a CADD score of 28. The variant was not predicted by the Missense3D tool to alter the protein structure, however. On the other hand, the variant was predicted to unmask a splice site inside exon 21 which may affect the mRNA stability and must then be explored in patient’s cells if expressed in leukocytes or fibroblasts. This was not possible, however. Pathogenic mutations in the *DMXL2* gene cause the autosomal dominant deafness type 71 (OMIM # 617605), and the autosomal recessive developmental and epileptic encephalopathy type 81 (OMIM # 618663) and polyendocrine-polyneuropathy syndrome (OMIM # 616113) [24–26]. We herein, potentially extended the phenotype of *DMXL2* mutations to include complex HSP. Details about the clinical presentations of the previous families with *DMXL2* variants are provided in the Supplementary material.

The variant NM_001145026.2:c.5893 C > A (p.Pro1965Thr) in *PTPRQ* was detected in two adult patients from family F85 who presented with congenital deafness and mutism but at different zygosity state. *PTPRQ* variants have been reports in AR and AD hearing loss with mildly delayed development (MIM # 617663 & 613391). This variant was also predicted as deleterious by Sift, Polyphen 2, MutationTaster, and Provean. It was absent in the gnomAD v2.1.1 database. We considered it as a VUS.

Details about the VUS identified in family F79 in HERC2 and ATP2B3 were provided in a previous report [19].

## Discussion

### Diagnosis yield

The Sudanese population is paradoxically characterized by a complex genetic structure and high consanguinity rates [6, 10]. The high level of homozygosity in our cohort was reflected by the predominance of homozygous recessive diseases (75%) and the detection of three established/possible founder variants. Two of these founder variants were in *ADAT3* and *PRUNE1* genes as we reported previously [19, 27]. The third possible founder variant, NM_024306.5:c.674 T > C (p.Leu225Pro), was in *FA2H* and was detected in two unrelated families, F61 and F68, that descended from different tribes in Kordofan province, western Sudan. Nevertheless, we also identified autosomal dominant and X-linked (hemizygous) conditions in several families.

Most of our families originated from the central parts of Sudan. This can be attributed either to differences in the accessibility to the health system and our collaborating clinics or genuine differences in the frequency of genetic diseases between central Sudan populations and other Sudanese populations. We favor the first explanation as other consanguinity-linked genetic diseases, such as sickle cell anemia, are common in non-central parts of the country [10].

All age groups were represented in our cohort, particularly those < 18 years, indicating the degree of care provided to this age group by their families. On the other hand, we have patients with childhood-onset diseases who were first examined after their forties (after decades of disease duration, > 40 years in two patients), epitomizing the long-term odysseys of patients with genetic diseases and underlining the importance of genetic diagnosis for patients’ and families’ satisfaction. Also, the percentages of males and females in our cohort were approximately equal, signifying the absence of gender-based inequalities in the accessibility of care and minimizing the contribution of X-linked dominant inheritance to SCDs in our cohort.

Previously, we screened 25 Sudanese families with HSP for mutations in 68 known HSP genes using NGS targeted gene panel [11]. We reached a genetic diagnosis in 28% of these cases [11], a diagnostic rate very similar to Portuguese [28] and European [12] patients. This last study, (ref. 12), showed that combining HSP panel with subsequent WES increased the diagnosis rate up to 50% when focusing on OMIM disease-related genes. WES used to further identify novel genes was shown to give a diagnostic yield of up to 75% [29]. In the current study, by using multiple genetic approaches, we identified disease-causing variants in known SCDs genes in 63–73% of the studied families. The overall diagnostic success rate if we consider our previous cohort (ref 11) is 52–59% (31–35/59 families). Furthermore, extending the analysis to all genes covered by the exome, we identified variants in novel candidate genes in seven out of the ten remaining families (see Tables 1, 2), potentially raising our diagnostic success rate ceiling to 92% instead of 73%. One of those seven novel causative genes has been reported [30] and the others are under validation and will be reported elsewhere (unpublished data). According to the results of our two studies, most of the major autosomal recessive SCDs genes are present in Sudan (*SACS*, *SPG11*, *FXN*) and some of the major dominant ones as well (e.g., *SCA3*), but there is no single major gene causing SCDs in Sudan. This might result from the position of Sudan in east Africa, at the frontiers between North Africa, the Middle East, and sub-Saharan Africa.

### Lessons for genetic diagnosis of SCD in Sudan

In five families, we could establish the diagnosis in only a portion of the patients or branches since the variants were not segregating in all patients, outlining the need to introduce into the analysis pipeline of Sudanese families an additional step that consists of analyzing the patients individually after excluding the variants shared by multiple patients from the same family.

WES outweighs NGS targeted gene panel in discovering new SCDs genes [4]. However, based on our experience with the Sudanese population, and the experience of others, exome sequencing also significantly outweighs NGS-targeted gene panels in diagnosing known SCDs phenotypes, particularly in complex phenotypes [31]. Furthermore, WES enables the extension of phenotypes previously associated with mutations in certain genes in contrast to conservative NGS-targeted gene panels that target only the phenotype of interest. For instance, we extended the phenotypes associated with mutations in *CCDC82* and *CCDC88C* in the current Sudanese cohort by using WES. *CCDC82* was reported previously to cause an intellectual disability syndrome [32, 33]. We expanded the *CCDC82*-linked phenotype to include spastic paraplegia [19]. Later, another report of a patient of Pakistani origin confirmed that spasticity is part of the *CCDC82*-linked syndrome [34]. Similarly, we expanded the presentation of heterozygous mutations in *CCDC88C* to include early-onset pure spastic paraplegia [35]. Before, heterozygous gain-of-function *CCDC88C* mutations were only associated with spinocerebellar ataxia SCA40 [36]. In this report we also potentially extended the phenotype of *DMXL2*- and *PTPRQ*-linked disorders to include complex HSP.

In our opinion, the higher diagnostic success rate of WES overrides its technical difficulties when compared to NGS-targeted gene panel upon studying diseases with overlapping phenotypes like SCDs, particularly when considering the increasing technical feasibility of WES [37]. However, WES is less efficient for rearrangement detection than panels of genes, usually optimized for such discovery, as discussed (ref, 12). An issue in SCDs is the detection of nucleotide repeat expansions that require independent specific techniques but there are improvements of some algorithm for such quest in WES data and in genome sequencing [38].

In conclusion, up-to-now, SCDs in Sudan are caused by multiple genes; none of them significantly predominate over the others. The use of multiple genetic approaches that included WES enhanced the diagnosis of known SCDs phenotypes and the potential discovery of new SCDs genes.

## Supplementary information

Supplementary Table

Supplementary material

## Data Availability

Some details of the participants, including family pedigrees and natural history, have been removed from this report to ensure anonymity and comply with the journal’s standards. Further information on segregation analysis and data supporting the findings of this study are available from the corresponding authors upon reasonable requests. All novel variants and VUS have been submitted to ClinVar (submission number SUB12076628).
