# Supplementary material for: Clinical phenotyping and genetic diagnosis of a large cohort of Sudanese families with hereditary spinocerebellar degenerations
Source: Eur J Hum Genet. 2023 Apr 3;32(10):1214–26. doi: 10.1038/s41431-023-01344-6 (PMC11499676; doi:10.1038/s41431-023-01344-6)
Supplement: Supplementary file 2 — Supplementary material [file 41431_2023_1344_MOESM2_ESM.pdf]

Clinical phenotyping and genetic diagnosis of a large cohort of Sudanese families with hereditary spinocerebellar degenerations (SCD).

Supplementary Material

Family F8

We identified the homozygous variant NM_001037633.2 (***SIL1***):c.767+1G>A on analyzing WES data of the two patients on absence of other convincing variants in other known SCD genes. This variant is predicted to disrupt the splice-site junction between exon eight and intron eight of the *SIL1* gene (TraP score 0.93). It was absent in the gnomAD v2.1.1 database. The variant was homozygous in patients and heterozygous in their parents.

Family F27

WES and downstream analysis identified the variants NM_000082.4(***ERCC8***):c.523T>C (p.Ser175Pro) and NM_000082.4:c.572_574del (p.Ala191del) *in trans* in patients F27-227, F27-228, and F27-229. The gene was known in Cockaine syndrome. We validated the variants and their segregation with the disease in family F27 using Sanger sequencing. Sift, Polyphen 2, MutationTaster, LRT, and Provean predicted the variant NM_000082.4:c.523T>C (p.Ser175Pro) as pathogenic. It had a CADD score of 27.7 and was absent from the gnomAD v.2.1.1 genomes and exomes databases. The variant NM_000082.4:c.572_574del (p.Ala191del) was also absent from the gnomAD v.2.1.1 database and predicted to delete a highly conserved amino acid.

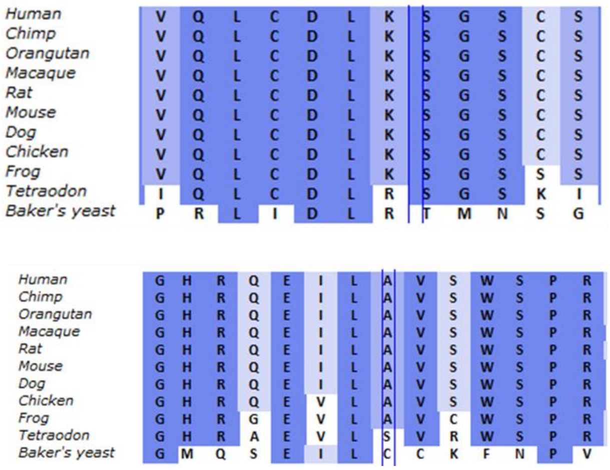

Figure 1. The conservation of the ERCC8 Ser175 (upper panel) and Ala191 (lower panel) across multiple species is shown.

Family F31

WES and downstream analysis identified the variant NM_003383.5(***VLDLR***):c.2144G>A (p.Cys715Tyr) (rs761330915) homozygous in patient F31-267 and heterozygous in her father, individual F31-264. This gene has already been involved in neurological disorders. We validated the variant and its segregation with the phenotype in family F31 using Sanger sequencing. The variant was absent from the gnomAD genomes database and had a low frequency in the gnomAD exomes database (0.00000398). Sift, Polyphen 2, MutationTaster, LRT, and Provean predicted the variant NM_003383.5:c.2144G>A as pathogenic. It had a CADD score of 29.1.

We excluded two potentially pathogenic heterozygous variants (Table 1) that passed our filters without analyzing their segregation by Sanger sequencing, because they were shared between patient F31-267 and her healthy father, individual F31-264.

Table 1. Variants that passed our filters and excluded before Sanger sequencing in family F31		
Variant	Frequency (gnomAD general population)	Phenotype
NM_001168272.2:c.6979C>T (p.Arg2327Trp) (heterozygous)	0.00002	SCA15 (OMIM # 606658) ; SCA29 (OMIM # 117360)
NM_015215.4:c.565G>A p.(Gly189Ser) (heterozygous)	0.0001	Cerebellar ataxia, nonprogressive, with mental retardation (OMIM # 611501)

In-silico VLDR protein modeling and analysis: VLDLR has three models in the protein data bank, but none include the Cys715 of the epidermal growth factor-like 3 (EGF-like 3) domain of VLDLR. We predicted the 3D structure of the VDLR EGF-like 3 domain using Phyre2 software (1). Subsequently, we analyzed the effects of the variant NM_003383.5:c.2144G>A (p.Cys715Tyr) on the predicted EFG-like 3 domain using Missense3D tool (2). The p.Cys715Tyr substitution triggered a clash alert in Missense3D and was predicted to break the disulfide bond between Cys715 and Cys734. The disruption of another disulfide bond, between Cys706 and Cys719, within the EGF-like 3 domain of VLDLR was predicted as disease-causing in two families of Omani origin harboring the variant NM_003383.5:c.2117G>T (p.Cys706Phe) (3). Several pathogenic VLDLR variants, including p.Cys706Phe, were reported to be aggregation-prone and cause ER stress (4). We did not examine the variant NM_003383.5:c.2144G>A (p.Cys715Tyr) for causing a similar cellular phenotype as cells from patients were not available.

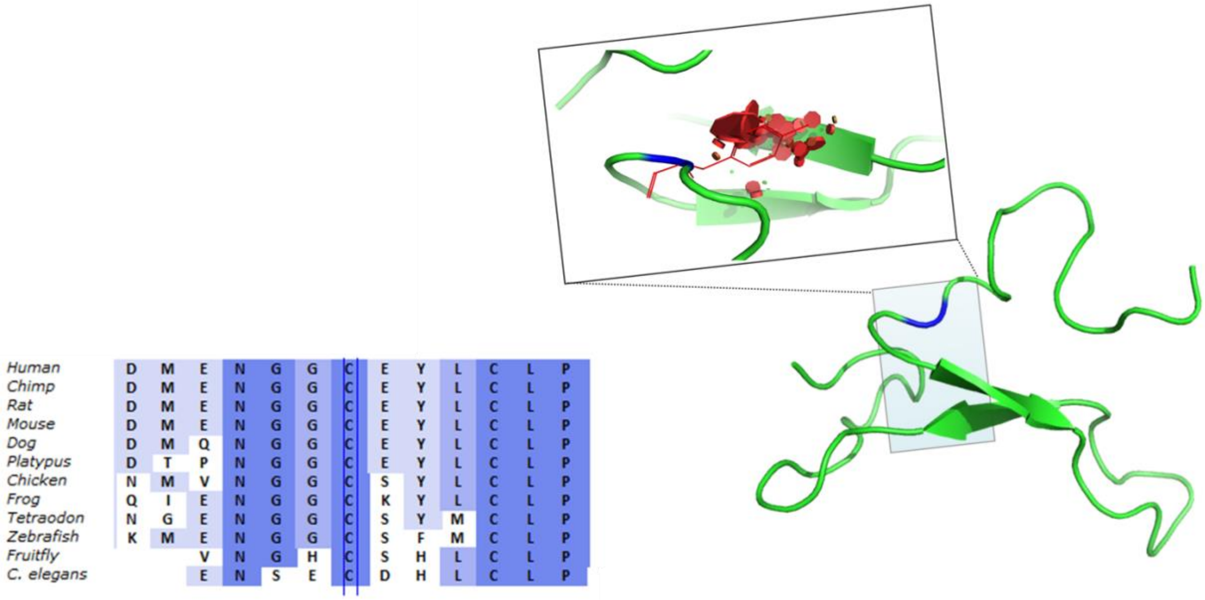

Figure 2. Location and conservation of VLDLR Cys715 and the structural consequences of the p.Cys715Tyr substitution. The predicted 3D structure of the VDLR EGF-like 3 domain and the predicted effect of the p.Cys715Tyr substitution. The wild-type residue is shown in blue and the mutant in red lines. The stearic clashes caused the substitution are shown as red polygons. The conservation of Cys715 across multiple species is shown.

Family F38

We suspected Friedreich ataxia on clinical grounds. We sent DNA from patient F38-318 for **Friedreich ataxia GAA expansion** screening, and it was returned positive. In parallel, the DNA of patients F38-318 and F38-329, was investigated using WES. The homozygous variant NM_001322042.2:c.2595-1G>C (rs587783739) in *MCPH1* was only identified in patient F38-329 and heterozygous in her parents. The variant rs587783739 was predicted to be a splice-acceptor variant by SpliceAI tool (score 0.98) and was classified as pathogenic in ClinVar (VCV000158863.5). Its frequency was 0.00001 in the gnomAD database. Pathogenic variants in *MCPH1* cause autosomal recessive primary microcephaly (OMIM # 251200). There were major differences between the known phenotype of *MCPH1* pathogenic mutations and the clinical phenotype in patient F38-329, particularly the absence of microcephaly and the late age-at-onset in our patient. We did not considered this variant as causative then.

Family F41

WES and downstream analysis identified the frame-shift variant NM_001042492.3:c.187_188del (***NF1***; p.Lys63GlufsTer3) in a heterozygous state in patient F41-351, but no candidate variant in her affected brother, patient F41-349. We validated the variant status in patient F41-351 and its absence in her family members using Sanger sequencing (including the parents) indicative of a *de novo* mutation. It was absent from the gnomAD v.2.1.1 database.

Family F49

Genomic DNA from patient F49-396 was screened for pathogenic DNA repeat expansions in the genes *ATXN1* (SCA1), *ATXN2* (SCA2), *ATXN3* (SCA3), *CACNA1A* (SCA6), *ATXN7* (SCA7), *TBP* (SCA17), and *ATN1* (DRPLA) using PCR-based approaches at the genetics department of the Pitié-Salpêtrière Hospital (Pr Eric Leguern). We detected a pathological 73+/- 3 CAG repeat expansion in the ***ATXN3*** gene and confirmed its segregation with the disease in the family.

Family F53

Targeted NGS panel screening and downstream analysis identified the variants NM_025137.4:c.2399del (***SPG11***; p.Tyr800Phefs*19) and NM_025137.4:c.6739_6742del (*SPG11*; p.Glu2247Leufs*14) (rs312262782) in trans in patients F53-417, F53-418, and F53-419. We validated the variants in the three patients using Sanger and validated their segregation with the disease in the family F53 in a compound heterozygous pattern of inheritance. The variant NM_025137.4:c.2399del was novel while NM_025137.4:c.6739_6742del was classified as pathogenic in the Clinvar database based on multiple submissions (VCV000041348.3) (https://www.ncbi.nlm.nih.gov/clinvar/variation/41348/) and had low frequency in the gnomAD v.2.1.1 exomes (0.0000517) and genomes (0.0000319) databases.

Family F54

WES and downstream analysis identified the homozygous variant NM_014855.3:c.1132G>A (***AP5Z1***; p.Gly378Arg) (rs777093701) in patient F54-427, but it was heterozygous in his affected sister, patient F54-426. *AP5Z1* variants are involved in SPG48. We validated the identified variant using Sanger sequencing. WES in patient F54-426 did not identify a second candidate variant

neither in *AP5Z1* nor in any other gene. NM_014855.3:c.1132G>A had a low frequency in the gnomAD v2.1.1 database (0.0001285) and not reported in the gnomAD v2.1.1 African population. Sift, Polyphen 2, MutationTaster, LRT, and Provean predicted the p.Gly378Arg substitution as pathogenic. It alters the highly conserved Gly378 (CADD score 32). Furthermore, NM_014855.3:c.1132G>A substitutes the last base before splice site in exon 9 of the *AP5Z1* mRNA and several tools predicted it as a splicing disruptive variant. A single submitter reported the variant NM_014855.3:c.1132G>A in association with SPG48 as a variant of uncertain significance in the ClinVar database (accession VCV000652848); however, no clinical details were provided.

Other variants excluded by segregation analysis are presented in Table 2.

Table 2. Variants that passed our filters and excluded by segregation analysis in family F54

Variant	Frequency (gnomAD general population)	Phenotype
NM_001349655.2:c.2353A>G (PIBF1; p.Lys785Glu) (homozygous)	0	Joubert syndrome 33 (OMIM # 617767)
NM_001008781.3:c.7378C>T (FAT3; p. Arg2460Trp) / NM_001008781.3:c.10972C>T (FAT3; p. Pro3658Ala) (compound heterozygous)	0.00001 / 0	Not associated with a disease

Family F57

Targeted NGS panel screening revealed the homozygous novel variant NM_014363.6:c.10444_10447del (***SACS***; p.Leu3482Glnfs*12) in the three patients. SACS mutations are well known in patients with spastic ataxia. We validated the variant in the three patients and its segregation with the disease in the family under an autosomal recessive model using Sanger sequencing. It is predicted to cause loss of the sacsin protein expression due to nonsense-mediated mRNA decay.

Family F59

Using a targeted approach on SPG genes, we identified the variant NM_001351171.2:c.175+1G>A (***NT5C2***; rs886037658) homozygous in the three patients from the family F59. NM_001351171.2:c.175+1G>A was predicted as a splice-site variant (Trap score 0.9) that alters a

highly conserved nucleotide (CADD score 35). It was absent in gnomAD v.2.1.1 genomes and exomes databases and was reported as pathogenic in the Clinvar database according to a single submission (accession VCV000100908.1). We confirmed the variant and its segregation in the 3 affected siblings of family F59 using Sanger sequencing.

Families F61 and F68

Targeted NGS panel screening identified the novel homozygous variant NM_024306.5:c.674T>C (***FA2H***; p.Leu225Pro) in patients F61-465, F61-467, F68-503, and F68-504 from families F61 and F68. *FA2H* mutations are involved in SPG35. We validated the variant and its segregation with the disease in the two families using Sanger sequencing. Sift, Polyphen 2, MutationTaster, LRT, and Provean predicted the variant NM_024306.5:c.674T>C as pathogenic. It had a CADD score of 24.6.

Family F62

WES and downstream analysis identified the homozygous splice-site variant NM_000124.4(***ERCC6***):c.4063-1G>C (rs766980240) in patients F62-470 and F62-471. The gene was known to be involved in Cockaine syndrome. We validated the variant in the two patients at the homozygous state and identified it in a heterozygous state in their parents and healthy brother using Sanger sequencing. The variant had a TraP score of 0.469 but was reported as likely pathogenic in the Clinvar database by two submitters (VCV000549960.6). It had a low frequency in the gnomAD v.2.1.1 exomes database, 0.0000201, and was absent in the gnomAD v.2.1.1 genomes database.

Family F63

We identified the NM_138422.4:c.430G>A (p.Val144Met) variant at the homozygous state in ***ADAT3*** in the affected case as reported (19).

Family F66

WES and downstream analysis of the DNA samples obtained from patients F66-492 and F66-493 identified the homozygous missense variant NM_001174116.3:c.5020A>C (***DMXL2***; p.Lys1674Gln) in the two affected brothers. Using Sanger sequencing, we validated the variant's

status in the two patients and identified it in a heterozygous state in their parents. It was absent in the gnomAD v.2.1.1 genome database and had a low frequency, 0.00000398, in the gnomAD v.2.1.1 exome database. Sift, Polyphen 2, MutationTaster, LRT, and Provean predicted the variant NM_001174116.3:c.5020A>C as pathogenic. It had a CADD score of 28 and was also predicted to unmask an exonic cryptic splice site. The affected amino-acid was strongly conserved up to flies.

DMXL2 encodes the α subunit of rabconnectin-3, a WD40-repeat (WDR)-containing protein abundantly expressed in the brain, particularly in the synaptic vesicles (5–7). Rabconnectin-3 also has a β subunit encoded by *WDR7* (6). The WDR-containing proteins are a large evolutionary conserved protein family involved in a broad range of biological processes (8,9). WDRs contain 4-16 repeats of 40-60 amino acids that end with aspartic acid and tryptophan (WD) important for their interactions with other proteins (9). More than 360 domains have been reported besides those 40-60 amino acid repeats in WDRs (8). WDRs are involved in the pathogenesis of multiple genetic human disorders; the majority thereof afflicts the nervous system (9).

Rabconnectin-3 participates in the regulation of Cav2.2 (CACNA1B) channels-mediated calcium entry (10). Pathogenic mutations in the *CACNA1B* gene cause neurodevelopmental disorder with seizures and nonepileptic hyperkinetic movements (OMIM # 618497) (11). Pathogenic mutations in the *DMXL2* gene cause the autosomal dominant deafness type 71 (OMIM # 617605), and the autosomal recessive developmental and epileptic encephalopathy type 81 (OMIM # 618663) and polyendocrine-polyneuropathy syndrome (OMIM # 616113) (12–14). The loss of *DMXL2* alters neuronal autophagy, development, and synaptic contacts (15).

Herein we identified the homozygous missense variant NM_001174116.3:c.5020A>C (p.Lys1674Gln) in two Sudanese siblings manifesting a neurodevelopmental disorder.

Before this study, six variants in the *DMXL2* gene were reported to cause recessive phenotypes (12,13,15). Interestingly, five variants were loss-of-function variants and one, NM_001174116.3:c.5135C>T (p.Ala1712Val), was a missense variant segregated *in trans* with a loss of function variant (12,13,15). The missense variant p.Lys1674Gln, which we identified in the two Sudanese patients, was not predicted by the Missense3D tool to alter the rabconnectin-3 structure. However, the variant p.Lys1674Gln alters a highly conserved amino acid and was predicted pathogenic by five prediction tools. Possibly, the p.Lys1674Gln variant disrupts important intra- or inter-proteins interactions or has structural effects not detected by our approach. On the other hand, the variant was predicted to unmask a splice site inside exon 21 which may

affect the mRNA stability and must then be explored in patient's cells if expressed in leukocytes or fibroblasts.

The clinical phenotypes linked to pathological recessive *DMXL2* mutations had a broad spectrum that includes neurological features in all the reported patients. The clinical phenotype of patients from the Sudanese family, the family F66, was milder compared to the other reported patients, except Senegalese patients. The phenotype in the older Sudanese patient, patient F66-492, was more severe than the Senegalese patients. The neurological manifestations in patients from the Senegalese family with the NM_001174116.3:c.5824_5838del (p.1942_1946del) variant appeared late compared to the other patients with *DMXL2* mutations. However, the Senegalese patients had marked endocrinological disturbances in the form of insulin-dependent diabetes mellitus, stunted growth, and incomplete puberty (12). These glandular abnormalities could be partially attributed to the effects of rabconnectin-3α on gonadotropin-releasing hormone (GnRH) secretion. Rabconnectin-3α participates in the morphological maturation of GnRH-secreting neurons and their responsiveness to the main GnRH secretagogue kisspeptin (16).

The nature of the variant, missense vs. loss-of-function, did not affect the severity of the *DMXL2*-linked phenotype. On the other hand, the reported clinical phenotype in the African patients was milder than in the non-African patients. The possible effects of background DNA variants and DNA modifiers on *DMXL2* were highlighted in a multi-generational Dutch family segregating a neurodevelopmental disorder and a heterozygous deletion of the *DMXL2* gene (17). However, the data about the *DMXL2* gene and its associated phenotypes is scanty. Identifying more patients with *DMXL2*-linked disorders will improve our understanding of these diseases and their modifiers.

Family F67

We analyzed the samples from patient F67-496 and her healthy father using WES. We identified the variant NM_152778.3:c.753A>G (***MFSD8***; rs1164872454) homozygous in the patient and heterozygous in her father and confirmed its status using Sanger sequencing. We also detected the variant in a heterozygous state in patient F65-496's mother. The variant NM_152778.3:c.753A>G (p.Glu251Glu) is a synonymous variant but predicted to alter the splicing of *MFSD8* (TraP score 0.96) and to cause skipping of exon 8 of the gene (according to Ex-SKIP tool). The variant rs1164872454 was absent from the gnomAD v2.1.1 database. We classified the variant

NM_152778.3:c.753A>G as a VUS according to the ACMG 2015 guidelines for interpreting sequence variants, due to lack of functional validation (18).

Family F68

See at family F61

Family F70

WES sequencing and downstream analysis revealed the homozygous splice-site variant NM_007055.4:c.1771-7C>G (***POLR3A***; rs201314157) in patient F70-513. It had a low frequency in the gnomAD v.2.1.1 exome and genome databases, 0.000223 and 0.000107, respectively, and was reported as pathogenic and likely pathogenic in the Clinvar database by four submitters and as a variant of uncertain significance by one submitter (accession VCV000449556.12). The variant NM_007055.4:c.1771-7C>G was validated in patient F70-513 using Sanger sequencing. It was absent in the other sampled members of her family, including her brother, patient F70-514. WES and downstream analysis of patient F70-514 did not identify any possible candidate variant. Segregation analysis excluded other variants that passed our filters in patients F70-513 and/or F70-514 (Table 3).

Table 3. Variants that passed our filters and excluded by segregation analysis in family F70

Patient	Variant	Frequency (gnomAD general population)	Phenotype
F70-514	NM_000701.8:c.1538G>A (ATP1A1; p.Arg513Lys) (heterozygous)	0	Autosomal dominant type 2 D axonal Charcot-Marie-Tooth disease (OMIM # 618036); autosomal dominant hypomagnesemia, seizures, and mental retardation type-2 syndrome (HOMGSMR2, OMIM # 618314); hereditary spastic paraplegia
F70-514	NM_152286.5:c.1865G>A (PNPLA7; p.Arg622His) (homozygous)	0	Not associated with a disease
F70-514	NM_000834.5:c.1253T>C (GRIN2B ; p.Ile418Thr) (heterozygous)	0	Developmental and epileptic encephalopathy 27 (OMIM # 616139)

F70-513	NM_198578.4:c.4112T>C (LRRK2; p.Ile1371Thr) (heterozygous)	0	Parkinson disease type 8 (OMIM # 607060)

Family F76

Targeted NGS screening panel and downstream analysis identified the homozygous variant NM_015214.3:c.985C>T (***DDHD2***; p.Arg329*) (rs201258800) in patients F76-547 and F76-548. We validated the variant's status in the patients and detected it in a similar state in their sister, patient F76-BB, using Sanger sequencing. We also validated NM_015214.3:c.985C>T segregation with the disease in the family F76 in an autosomal recessive pattern using Sanger sequencing. The variant was absent in the gnomAD v.2.1.1 genomes database and had a low frequency in the gnomAD exomes database (0.0000159). It was classified as pathogenic in the Clinvar database based on multiple submissions (VCV000452548.4) (https://www.ncbi.nlm.nih.gov/clinvar/variation/452548/).

Family F78

Targeted NGS panel and downstream analysis identified the novel frame-shift variant NM_015346.4:c.1254dup (***ZFYVE26***; p.Cys419Valfs*61) in patient F78-557. We validated the variant's status in patient F78-557 and identified a similar genotype in affected sister F78-AA using Sanger sequencing. Sanger sequencing also confirmed the variant's segregation with the disease under an autosomal recessive inheritance pattern in the family F78 (parents were heterozygous).

Family F79

We identified 2 interesting variants AR homozygous (NM_004667.6:c.10855C>T; p.Pro3619Ser) in ***HERC2***) and X-linked (NM_021949.3:c.2086C>T; p.Arg696Cys in ATP2B3). Their involvement in the disease are discussed (19).

Family F80

WES and downstream analysis revealed the frame-shift variant NM_001134373.3:c.629del (***NT5C2***; p.Tyr210Serfs*17) homozygous in patient F80-573 but absent in her affected sister, patient F80-572. The results of the WES analysis were validated using Sanger sequencing. Sanger

sequencing also detected the variant as heterozygous in her parents. WES data of patient F80-572 and the downstream analysis did not identify any candidate variant.

Family F81

We identified the the homozygous NM_001318736.2:c.535C>T (p.Arg179*) in ***CCDC82*** as discussed (19).

Family F82

We identified the X-linked NM_031407.7:c.12639G>A (p.Met4213Ile) variant in ***HUWE1*** as reported elsewhere (19).

Family F83

We identified the ***CCDC88C*** NM_001080414.4:c.1993G>A (p.Glu665Lys) as causative at the heterozygous state as discussed elsewhere (20).

Family F84

WES and downstream analysis revealed the homozygous nonsense variant NM_020533.3:c.514C>T (***MCOLN1***; p.Arg172*) (rs797044824) in the two affected siblings. We detected the variant in a heterozygous state in the parents and validated its presence in patients using Sanger sequencing. The variant NM_020533.3:c.514C>T was absent in the gnomAD v.2.1.1 genomes and exomes databases and was labeled as likely pathogenic in the Clinvar database (accession VCV000208030.1).

Family F85

This family presented a pseudodominant inheritance. Analysis of patient 2056's exome data identified the homozygous variant NM_001145026.2:c.5893C>A (***PTPRQ***; p.Pro1965Thr). The variant was heterozygous in his affected child 2059 and in the grandfather. It was absent in the gnomAD v2.1.1 database and predicted as pathogenic by Sift, Polyphen-2, MutationTaster, and Provean, and neutral by LRT. It affected a well conserved amino-acid (Figure 3).

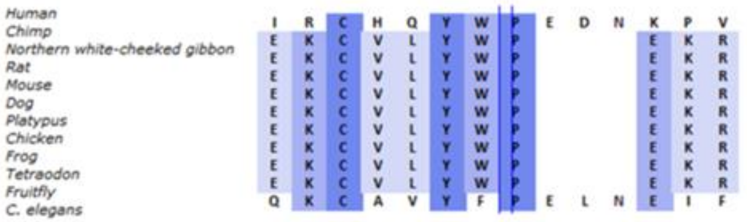

Figure 3. Pro1965 is conserved from humans to c.elegans.

Family FM3

We identified the homozygous and pathogenic variant NM_021222.3:c.132+2T>C (splice-site) in ***PRUNE1*** as described (21).

Family FM6

We identified the compound herterozygous variants NM_018122.5:c.1762C>G (p.Leu588Val) / NM_018122.5:c.563G>A (p.Arg188Gln) predicted likely pathogenic in the ***DARS2*** gene as described (22).

References

1. Kelley LA, Mezulis S, Yates CM, Wass MN, Sternberg MJE. The Phyre2 web portal for protein modeling, prediction and analysis. Nat Protoc [Internet]. 2015 Jun 30 [cited 2020 Nov 7];10(6):845–58. Available from: https://pubmed.ncbi.nlm.nih.gov/25950237/
2. Ittisoponpisan S, Islam SA, Khanna T, Alhuzimi E, David A, Sternberg MJE. Can Predicted Protein 3D Structures Provide Reliable Insights into whether Missense Variants Are Disease Associated? J Mol Biol [Internet]. 2019 May 17 [cited 2021 Jan 5];431(11):2197–212. Available from: https://pubmed.ncbi.nlm.nih.gov/30995449/
3. Ali BR, Silhavy JL, Gleeson MJ, Gleeson JG, Al-Gazali L. A missense founder mutation in VLDLR is associated with Dysequilibrium Syndrome without quadrupedal locomotion. BMC Med Genet [Internet]. 2012 Sep 14 [cited 2021 Jan 15];13:80. Available from: /pmc/articles/PMC3495048/?report=abstract
4. Kizhakkedath P, John A, Al-Gazali L, Ali BR. Degradation routes of trafficking-defective VLDLR mutants associated with Dysequilibrium syndrome. Sci Rep [Internet]. 2018 Dec 1 [cited 2021 Jan 15];8(1). Available from: /pmc/articles/PMC5785505/?report=abstract

5. Nagano F, Kawabe H, Nakanishi H, Shinohara M, Deguchi-Tawarada M, Takeuchi M, et al. Rabconnectin-3, a novel protein that binds both GDP/GTP exchange protein and GTPase-activating protein for Rab3 small G protein family. J Biol Chem [Internet]. 2002 Mar 22 [cited 2021 Jan 3];277(12):9629–32. Available from: https://pubmed.ncbi.nlm.nih.gov/11809763/
6. Kawabe H, Sakisaka T, Yasumi M, Shingai T, Izumi G, Nagano F, et al. A novel rabconnectin-3-binding protein that directly binds a GDP/GTP exchange protein for Rab3A small G protein implicated in Ca2+-dependent exocytosis of neurotransmitter. Genes to Cells [Internet]. 2003 Jun 1 [cited 2021 Jan 3];8(6):537–46. Available from: https://pubmed.ncbi.nlm.nih.gov/12786944/
7. Kannan M, Bayam E, Wagner C, Rinaldi B, Kretz PF, Tilly P, et al. WD40-repeat 47, a microtubule-associated protein, is essential for brain development and autophagy. Proc Natl Acad Sci U S A [Internet]. 2017 Oct 31 [cited 2021 Jan 4];114(44):E9308–17. Available from: https://pubmed.ncbi.nlm.nih.gov/29078390/
8. Schapira M, Tyers M, Torrent M, Arrowsmith CH. WD40 repeat domain proteins: A novel target class? [Internet]. Vol. 16, Nature Reviews Drug Discovery. Nature Publishing Group; 2017 [cited 2021 Jan 4]. p. 773–86. Available from: /pmc/articles/PMC5975957/?report=abstract
9. Kim Y, Kim SH. WD40-Repeat Proteins in Ciliopathies and Congenital Disorders of Endocrine System [Internet]. Vol. 35, Endocrinology and Metabolism. Korean Endocrine Society; 2020 [cited 2021 Jan 4]. p. 494–506. Available from: /pmc/articles/PMC7520596/?report=abstract
10. Gandini MA, Souza IA, Fan J, Li K, Wang D, Zamponi GW. Interactions of Rabconnectin-3 with Cav2 calcium channels. Mol Brain [Internet]. 2019 Jun 28 [cited 2021 Jan 3];12(1). Available from: /pmc/articles/PMC6599304/?report=abstract
11. Gorman KM, Meyer E, Grozeva D, Spinelli E, McTague A, Sanchis-Juan A, et al. Bi-allelic Loss-of-Function CACNA1B Mutations in Progressive Epilepsy-Dyskinesia. Am J Hum Genet [Internet]. 2019 May 2 [cited 2021 Jan 3];104(5):948–56. Available from: https://pubmed.ncbi.nlm.nih.gov/30982612/
12. Tata B, Huijbregts L, Jacquier S, Csaba Z, Genin E, Meyer V, et al. Haploinsufficiency of Dmxl2, Encoding a Synaptic Protein, Causes Infertility Associated with a Loss of GnRH

Neurons in Mouse. PLoS Biol [Internet]. 2014 [cited 2021 Jan 3];12(9). Available from: https://pubmed.ncbi.nlm.nih.gov/25248098/

13. Maddirevula S, Alzahrani F, Al-Owain M, Al Muhaizea MA, Kayyali HR, AlHashem A, et al. Autozygome and high throughput confirmation of disease genes candidacy. Genet Med [Internet]. 2019 Mar 1 [cited 2021 Jan 3];21(3):736–42. Available from: https://pubmed.ncbi.nlm.nih.gov/30237576/

14. Chen DY, Liu XF, Lin XJ, Zhang D, Chai YC, Yu DH, et al. A dominant variant in DMXL2 is linked to nonsyndromic hearing loss. Genet Med [Internet]. 2017 May 1 [cited 2021 Jan 3];19(5):553–8. Available from: https://pubmed.ncbi.nlm.nih.gov/27657680/

15. Esposito A, Falace A, Wagner M, Gal M, Mei D, Conti V, et al. Biallelic DMXL2 mutations impair autophagy and cause Ohtahara syndrome with progressive course. Brain [Internet]. 2019 Dec 1 [cited 2021 Jan 3];142(12):3876–91. Available from: https://pubmed.ncbi.nlm.nih.gov/31688942/

16. Tata BK, Harbulot C, Csaba Z, Peineau S, Jacquier S, De Roux N. Rabconnectin-3α is required for the morphological maturation of GnRH neurons and kisspeptin responsiveness. Sci Rep [Internet]. 2017 Feb 17 [cited 2021 Jan 4];7. Available from: https://pubmed.ncbi.nlm.nih.gov/28209974/

17. Costain G, Walker S, Argiropoulos B, Baribeau DA, Bassett AS, Boot E, et al. Rare copy number variations affecting the synaptic gene DMXL2 in neurodevelopmental disorders. J Neurodev Disord [Internet]. 2019 Feb 7 [cited 2021 Jan 4];11(1). Available from: https://pubmed.ncbi.nlm.nih.gov/30732576/

18. Richards S, Aziz N, Bale S, Bick D, Das S, Gastier-Foster J, et al. Standards and guidelines for the interpretation of sequence variants: A joint consensus recommendation of the American College of Medical Genetics and Genomics and the Association for Molecular Pathology. Genet Med [Internet]. 2015 May 8 [cited 2020 Dec 19];17(5):405–24. Available from: https://pubmed.ncbi.nlm.nih.gov/25741868/

19 Yahia A, Ayed I ben, Hamed AA, Mohammed IN, Elseed MA, Bakhiet AM, et al. Genetic diagnosis in Sudanese and Tunisian families with syndromic intellectual disability through exome sequencing. Ann Hum Genet. 2022;86:181-194.

20 Yahia A, Chen ZS, Ahmed AE, Emad S, Adil R, Abubaker R, et al. A heterozygous mutation in the CCDC88C gene likely causes early-onset pure hereditary spastic paraplegia: a case

report. BMC Neurol. 2021;21(1):78. Available from: https://bmcneurol.biomedcentral.com/articles/10.1186/s12883-021-02113-y

21 Koko M, Yahia A, Elsayed LE, Hamed AA, Mohammed IN, Elseed MA, et al. An identical-by-descent novel splice-donor variant in PRUNE1 causes a neurodevelopmental syndrome with prominent dystonia in two consanguineous Sudanese families. Ann Hum Genet. 2021;85:186-195. Available from: https://onlinelibrary.wiley.com/doi/10.1111/ahg.12437

22 Yahia A, Elsayed L, Babai A, Salih MA, El-Sadig SM, Amin M, et al. Intra-familial phenotypic heterogeneity in a Sudanese family with DARS2-related leukoencephalopathy, brainstem and spinal cord involvement and lactate elevation: a case report. BMC Neurol. 2018;18(1):175. Available from: https://www.ncbi.nlm.nih.gov/pubmed/30352563
